# Examining the relationship between academic stress and motivation toward physical education within a semester: A two-wave study with Chinese secondary school students

**DOI:** 10.3389/fpsyg.2022.965690

**Published:** 2022-09-16

**Authors:** Menglu Yang, Carme Viladrich, Jaume Cruz

**Affiliations:** ^1^School of Psychology and Cognitive Science, East China Normal University, Shanghai, China; ^2^Department of Psychobiology, Methodology and Health Science, Universitat Autònoma de Barcelona, Barcelona, Spain; ^3^Department of Basic, Developmental and Educational Psychology, Universitat Autònoma de Barcelona, Barcelona, Spain

**Keywords:** academic stress, adolescent, motivation, physical education, self-determination theory

## Abstract

The present study aimed to investigate the relationship between academic stress and motivation toward physical education (PE) through a longitudinal design with cross-lagged panel analyses. A sample of 556 Chinese secondary school students participated in the research and completed Perceived Locus of Causality Scale and Educational Stress Scale for Adolescents at the beginning of the semester and 3 months later. The results demonstrated that academic stress factors were positively related to less self-determined motivations except that worry about grades was positively related to more self-determined motivations within each time point. In addition, we found that academic stress negatively predicted more self-determined motivations but positively predicted less self-determined motivations, whereas worry about grades negatively predicted amotivation 3 months later. Meanwhile, the influence of amotivation on despondency was also found. These results suggest that academic stress can obstruct students’ participation in PE through an impact on self-determined motivation. Our findings also indicate that self-determined students in PE will seek academic achievement as well, which in turn improves students’ academic status.

## Introduction

Academic stress, which largely comes from work overload, the amount of material to learn, and the need to achieve a high grade, becomes a critical problem during adolescence (Leung et al., 2010). In addition to the direct impact of stress on physical and mental health among adolescents, researchers also suggested the indirect impact of stress on health through poor health behaviors (Michels et al., 2015). Furthermore, participation in physical education (PE) has been found to be obstructed by the academic burden (Mowling et al., 2004; Back, 2015). Especially in Asian countries, the most important barriers to PE participation come from a prioritized emphasis on academic achievement of principal subjects, like mathematics and literature (Zhu et al., 2017).

Among various types of stress, academic stress may be the most salient for adolescents, which is highly related to competitive academic examinations, such as university entrance examinations and high school entrance examinations (Jun and Choi, 2015). Consistent with the transactional model of psychosocial stress proposed by Lazarus (1966), the level of perceived academic stress is determined not only by the number of academic stressors but also by how students interpret these stressors (Chua et al., 2018). Therefore, Sun et al. (2013) defined academic stress as subjective psychological distress from multiple aspects of academic learning, rather than a sum of stressors. The sources of academic stress can be originated from the heavy burden of homework, negative attitudes toward learning, such as loss of interest and difficulties in learning, etc (e.g., Wunsch et al., 2017). Meanwhile, the academic expectations from students and significant others, like parents and teachers, have also been demonstrated to be important factors of academic stress (Sun et al., 2011). Academic stress occurs when academic demands exceed a student’s perceived ability to cope with them (Wilks, 2008). As the burden of academic material varies within a semester, the academic stress may also change over time correspondingly (Xiang et al., 2019).

In previous studies, academic stress is associated with negative psychological consequences such as unpleasant emotional states, depression, tearfulness, even self-harm, and suicidal feelings in some cases (e.g., Lotz and Sparfeldt, 2017; Soares and Woods, 2020). Besides, students with a high level of stress also presented poor academic achievement, low self-efficacy, and low PE participation (Moeini et al., 2008; Park et al., 2020). Meanwhile, some researchers suggested that academic stress may not necessarily result in negative outcomes (Sang et al., 2018; Ye et al., 2019). Students’ responses to stress differ by their abilities and beliefs. Therefore, if students view academic challenges as opportunities and exert effort to meet the challenges, the academic stress may result in a positive impact (Sang et al., 2018).

### Motivation toward physical education

Regular engagement in Physical activity (PA) has been found to be related to the reduction of stress (Ki et al., 2019). As an important part of school education, one main goal of PE is to provide adolescents with PA during the course (Sallis et al., 2012). Therefore, participation in PE has been proposed as an alternative for reducing the life stress levels of adolescents. Despite the benefits of PE, the common view that PA increases at the cost of decreased time on principal subjects lead to a tendency of low participation in PE as well as a heavy academic burden (Wang, 2017). Nevertheless, several studies showed that PE and other PA programs did not result in negative academic outcomes, but were positively associated with academic outcomes, such as test scores, grades, and reduced academic stress (e.g., Sallis et al., 2012; Back, 2015; Shen, 2017). The influence of participation in PE on academic status may exert through physical and psychological fitness. Such influence may also be explained by the fact that students with high motivation to participate in PE will also be motivated to strive for achievement in academic subjects (Torrijos-Niño et al., 2014).

In order to increase active learning time in PE, Self-Determination Theory (Deci and Ryan, 1985) has been widely employed to explore students’ behavior, cognition, and affect in PE. According to Self-Determination Theory (Ryan and Deci, 2017), different forms of motivation situate along a self-determination continuum from more self-determined to less self-determined: intrinsic motivation, identified regulation, introjected regulation, external regulation, and amotivation. Sheldon and Elliot (1998) further proposed autonomous motivation which consists of more self-determined forms of motivation (i.e., intrinsic motivation and identified regulation), and controlled motivation which consists of less self-determined forms of motivation (i.e., introjected regulation and external regulation). Students with autonomous motivation will experience positive cognitive, affective, and behavioral consequences, such as vitality and enjoyment (e.g., Vlachopoulos, 2012; Gråstén and Watt, 2017). On the other side, controlled motivation and amotivation have been found to be related to negative outcomes, such as boredom, low engagement, and fear of exams (e.g., Haerens et al., 2010; Aelterman et al., 2012).

#### Academic stress and motivation toward physical education

Various studies have found relationships between academic stress and academic motivation (e.g., Liu, 2015; Rubach and Bonanati, 2021). Academic stress was found to negatively predict academic motivation, while high autonomous academic motivation led to a decrease in academic stress (Rubach and Bonanati, 2021). However, limited research has examined such reciprocal effects of students’ stress and motivation across contexts (e.g., Back, 2015). As suggested by Ryan and Deci (2017), students’ motivation in certain situations can be generalized to wider school experience. For instance, intrinsic motivation experienced in garden work could improve students’ motivation and experience in regular classroom courses (Skinner et al., 2012). Since PE has been considered to be an effective method to reduce academic stress (Ki et al., 2019), the autonomous motivation toward PE, as a predictor of participation, may also serve as an important factor to relieve academic stress.

Previous studies have found the positive effect of motivation toward PE on students’ perceived stress and academic attitudes (Back, 2015; Park et al., 2020). On the other side, a study conducted on student-athletes found that as perceived stress increased, students’ motivation for sports participation declined (Holden et al., 2019). In addition, factors related to testing stress, such as performance grading and test criteria, were also found to influence students’ autonomous motivation toward PE (Krijgsman et al., 2017; Haerens et al., 2019). Based on the results of these studies, we proposed a reciprocal relationship between stress and motivation across contexts.

### Academic stress and physical education in China

Although academic performance is a major source of stress among adolescents worldwide, this phenomenon seems to be more pronounced in Asian countries (Liu and Lu, 2012). Particularly, students from East-Asian countries, such as China, tend to spend more time taking classes and doing homework and perceive more academic stress than students from Western countries do (Ye et al., 2019). Due to highly valued academic achievement in traditional Chinese culture, academic performance has become a concern among Chinese adolescents and their parents as well as educators and administrators, which results in exam-oriented education, excessive stress, orderly school disciplinary climate, and lack of academic motivation (Sun et al., 2013; Ye et al., 2019; Ning, 2020).

A view commonly expressed in China is that sport requires little intellect (Jones, 1999). Although PE is recognized as a necessary part of education with the objective of health and fitness development which requires students to participate in PE and other school exercises for at least 1 hour every day, PE is not a subject of entrance examinations. Hence, considerable emphasis on academic success and university education has resulted in the negligence of PE in middle schools and even in primary schools. In a study of students from Shanghai (Zhu et al., 2017), the academic burden was found to be the primary reason for not having sufficient PA. Those with academic burdens were less likely to meet the PA guidelines but reported longer homework time. Consequently, Chinese adolescents showed increased physical inactivity and sedentary behavior in recent years and more than 85% of them failed to meet the guideline of about 60 min daily PA (Mowling et al., 2004; Lu et al., 2017; Liu et al., 2019).

### The present study

In consideration of the fact that Chinese adolescents experience high academic stress and limited participation in PA, we consider that the Chinese educational context could serve as an interesting context to explore how to reduce academic stress levels and promote PA participation. Using data from secondary school students in Shanghai who have one of the highest academic burdens and heaviest homework loads, we aimed to examine how academic stress levels and self-determined motivation toward PE change over time. Because of the lacking research on the relationship between stress and motivation across contexts, we also aimed to examine the relationship between academic stress and motivation toward PE to explore whether the influence of motivation-related factors can be generalized to other contexts. Based on the existing literature, we hypothesized that (1) both academic stress and motivation would change over time, and specifically, academic stress would decrease, and motivation would increase; (2) academic stress would relate negatively to self-determined motivation; (3) academic stress would predict a decrease in self-determined motivation toward PE, while self-determined motivation toward PE would predict a reduction in academic stress. We will test these hypotheses in turn after checking the measurement model for stress and motivation.

## Materials and methods

### Design

The current study applies a non-experimental longitudinal design. We conducted two evaluations of academic stress and motivation toward PE in single group of secondary school students across 3 months within the semester. Exclusion criteria for participants included the presence of cognitive deficits or significant sociocultural differences.

### Participants

A sample of 556 students (47.84% female) from Year 6 to 8 of four schools in average socio-cultural areas in Shanghai, with a mean age of 12.55 years (SD = 0.78, range: 10–15) participated in the study at the beginning of the fall semester and 3 months later. Among them, 463 students completed the questionnaires at both data collections, 34 students were absent at the first data collection, and 49 students were absent at the second data collection.

### Instruments

#### Academic stress

We used Educational Stress Scale for Adolescents (Sun et al., 2011), which was originally developed and administrated among Chinese adolescents, to measure students’ academic stress. The scale contains five subscales, 4-item subscale pressure from study (e.g., “I feel a lot of pressure in my daily studying”), 3-item subscale workload (e.g., “I feel there is too much homework”), 3-item subscale worry about grades (e.g., “I feel that I have disappointed my teacher when my test/exam results are not ideal”), 3-item subscale self-expectation (e.g., “I feel stressed when I do not live up to my own standards”), and 3-item subscale despondency (e.g., “I am very dissatisfied with my academic grades”). Students were asked to respond on a five-point Likert scale ranging from 1 (strongly disagree) to 5 (strongly agree). See validity and reliability for the present sample in the Section “Results.”

#### Motivation toward physical education

We administrated the adapted Chinese version of the Perceived Locus of Causality Scale (Goudas et al., 1994; Yang et al., 2019) to assess motivation toward PE. We measured three factors: autonomous motivation, formed by 4-item subscale intrinsic motivation (e.g., “because PE is fun.”) and 3-item subscale identified regulation (e.g., “because I want to learn sports skills.”), 3-item subscale controlled motivation, formed by introjected regulation (e.g., “because I would feel bad about myself if I didn’t.”) and 3-item subscale external regulation (e.g., “because that’s the rule.”), and 4-item subscale amotivation (e.g., “but I really don’t know why.”). Preceded by the stem “I participate in PE,” students were asked to respond on a five-point Likert scale ranging from 1 (strongly disagree) to 5 (strongly agree). See validity and reliability for the present sample in the Section “Results.”

### Procedure

After obtaining permission from the participating schools and ethical approval from the research ethics committee of the first author’ institution, we contacted teachers and/or directors of the PE department to approach the students for participation in the study. We informed students and their parents of the purpose of the research, the confidential procedure, and the voluntary participation to obtain consent from both sides. With the help of school teachers, we administrated the questionnaires before a PE course. Once students decided to participate in the study voluntarily, they began responding to questionnaires, which last between 10 and 25 min. To enable data matching over time without the need for names, we used ID code lists. All the procedures were following the ethical standards of the institutional human research committee.

### Data analysis

We examined first the measurement model using the weighted least squares means and variance adjusted estimator and the internal consistency of each subscale with Cronbach’s alpha and non-linear reliability coefficients (Green and Yang, 2009; Appelbaum et al., 2018). We used Comparative Fit Index (CFI) and Tucker-Lewis Index (TLI) >0.90 and Root Mean Squared Error of Approximation (RMSEA) <0.08, as criteria indicative of an acceptable model fit for quantitative data and CFI and TLI >0.95 and RMSEA <0.06 as criteria indicative of a good model fit (Kline, 2016). Since including all items and factors simultaneously in panel models requires a large sample size to produce stable estimates (Kline, 2016), we used the composite scores of factors for further analyses intended to test the main hypothesis. We compared the factors between two collections with paired *t*-test to explore the changes in academic stress and motivation toward PE over time and calculated Pearson correlation coefficients to examine the relationship among the factors within each time point.

Finally, we conducted the cross-lagged panel analyses to examine the relationship between academic stress and motivation toward PE over time, using the maximum likelihood estimator (Newsom, 2015). We tested and compared four models: Model 1 with autoregressive paths between two-time points; Model 2 with autoregressive paths and cross-lagged effects of academic stress on motivation; Model 3 with autoregressive paths and cross-lagged effects of motivation on academic stress; Model 4 with all autoregressive and cross-lagged effects paths. In order to choose the best-fitting model, we compared the models with cross-lagged effects (i.e., Model 2, Model 3, and Model 4) with the stability model with only the autoregressive paths (i.e., Model 1) through the χ^2^ difference test.

## Results

Missing data were infrequent (3.52% at the first collection; 3.22% at the second collection), which did not require special missing data treatment (Graham, 2009). The values of skewness of all items are between −1.11 and 1.15, and the values of kurtosis of all items are 2.28 and 4.03 which indicated a normal distribution in general. CFI and TLI of the measurement model were above 0.90 and RMSEA was lower than 0.06, which supported the structure of the administrated scales (see the first line in Table 1). In addition, Cronbach’s alpha coefficients of all subscales were higher than 0.7 as well as most of the non-linear internal consistency coefficients except that of despondency close to 0.7, which supported the internal consistency of the subscales of academic stress and motivation toward PE (Hair et al., 2018). See Table 2.

**TABLE 1:** Model fit indices.

Model	χ 2	*df*	CFI	TLI	RMSEA	90% CI RMSEA
Measurement model	4461.66	1959	0.922	0.915	0.049	[0.047, 0.050]
Cross-lagged models						
Model 1 with autoregressive paths	107.95	56	0.972	0.953	0.049	[0.035, 0.062]
Model 2 with autoregressive paths and cross-lagged effects of academic stress on motivation	84.39	41	0.976	0.947	0.052	[0.036, 0.068]
Model 3 with autoregressive paths and cross-lagged effects of motivation on academic stress	83.27	41	0.977	0.948	0.051	[0.035, 0.067]
Model 4 with all autoregressive and cross-lagged effects paths	59.44	26	0.982	0.935	0.057	[0.038, 0.077]

df, degrees of freedom; CFI, Comparative Fit Index; TLI, Tucker–Lewis Index; RMSEA, Root Mean Squared Error of Approximation; CI, Confidence Interval.

**TABLE 2:** Descriptive statistics and internal consistency coefficients.

	First collection				Second collection					
	*M*	*SD*	α	ω	*M*	*SD*	α	ω	Δ*M*	Range
Pressure from study	2.84	1.03	0.85	0.83	2.63	1.11	0.91	0.89	−0.21***	1–5
Workload	2.63	1.14	0.90	0.91	2.25	1.06	0.91	0.92	−0.37***	1–5
Worry about grades	3.75	1.01	0.85	0.84	3.70	1.06	0.86	0.86	−0.05	1–5
Self-expectation	2.96	1.02	0.74	0.71	2.88	1.07	0.79	0.76	−0.08	1–5
Despondency	2.34	0.91	0.70	0.67	2.47	0.96	0.71	0.70	0.13**	1–5
Autonomous motivation	3.98	0.90	0.89	0.93	4.10	0.95	0.92	0.95	0.12**	1–5
Controlled motivation	2.67	0.91	0.72	0.79	2.46	0.90	0.73	0.81	−0.21***	1–5
Amotivation	1.89	0.92	0.86	0.84	1.76	0.89	0.87	0.85	−0.13**	1–5

α = Cronbach’s alpha coefficient; ω = non-linear reliability coefficient; Δ = change from first collection to second collection. **p < 0.01; ***p < 0.001.

Regarding the difference between the two data collections, pressure from study, workload, controlled motivation and amotivation decreased significantly while despondency and autonomous motivation increased significantly from the first collection to the second collection (see Table 2, penultimate column).

Table 3 presents the correlation coefficients between variables at the first time point and the second time point separately. In both data collections, we found positive intercorrelations among academic stress factors. With regards to motivation toward PE, autonomous motivation was negatively related to controlled motivation and amotivation, while controlled motivation and amotivation were positively intercorrelated with each other. In respect of the relationship between academic stress and motivation, pressure from study, workload, and despondency were negatively associated with autonomous motivation and positively associated with controlled motivation and amotivation. Worry about grades was positively related to both autonomous motivation and controlled motivation but not significantly related to amotivation. Self-expectation was positively correlated with controlled motivation and amotivation but not significantly correlated with autonomous motivation.

**TABLE 3:** Correlations among academic stress and motivation toward physical education (PE) (first collection below diagonal and second collection above diagonal).

	1	2	3	4	5	6	7	8
1. Pressure from study	—	0.77***	0.36***	0.46***	0.65***	−0.19***	0.40***	0.41***
2. Workload	0.71***	—	0.16***	0.31***	0.54***	−0.28***	0.37***	0.41***
3. Worry about grades	0.22***	0.06	—	0.49***	0.30***	0.16***	0.31***	0.08
4. Self-expectation	0.37***	0.20***	0.38***	—	0.46***	−0.01	0.27***	0.19***
5. Despondency	0.48***	0.35***	0.10*	0.31***	—	−0.25***	0.36***	0.48***
6. Autonomous motivation	−0.15**	−0.20***	0.22***	0.08	−0.15**	—	−0.12**	−0.51***
7. Controlled motivation	0.40***	0.37***	0.25***	0.25***	0.18***	−0.06	—	0.45***
8. Amotivation	0.32***	0.32***	−0.09	0.06	0.22***	−0.54***	0.39***	—

*p < 0.05; **p < 0.01; ***p < 0.001.

**FIGURE 1:**
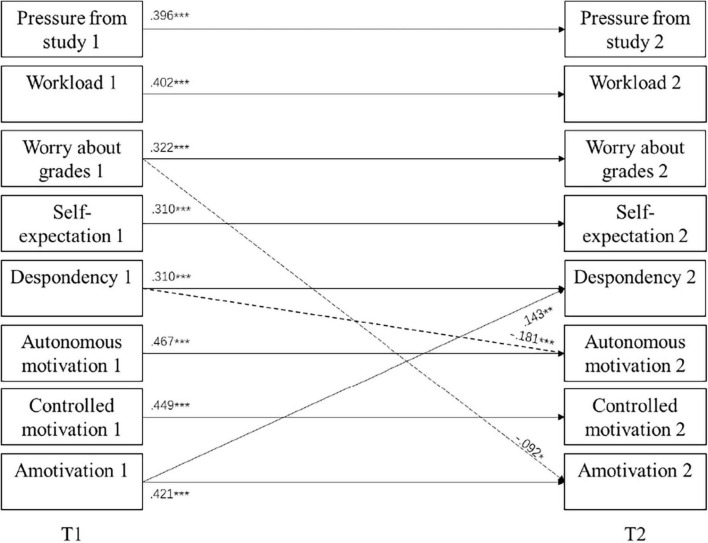
Cross-lagged model of the relationship between academic stress and motivation toward physical education (PE). Dashed lines depict the negative regression. *p < 0.05; **p < 0.01; ***p < 0.001.

Table 1 also presents the fit indices of cross-lagged models between two time points. All the models fit well with the data. Model 1 showed high stability of academic stress and motivation toward PE across 3 months with significant standardized autoregression coefficients ranging from 0.30 to 0.46. In Model 2, both the path from despondency to autonomous motivation (β = −0.18, *p* = < 0.001) and the path from worry about grades to amotivation (β = −0.09, *p* = 0.041) were significant. In Model 3, only the effect of amotivation on despondency (β = 0.13, *p* = 0.016) was significant. As can be seen in Figure 1, in the reciprocal model, worry about grades negatively predicted amotivation (β = −0.09, *p* = 0.040), and despondency negatively predicted autonomous motivation (β = −0.18, *p* < 0.001), while amotivation positively predicted despondency (β = 0.14, *p* = 0.010). Comparing all the models, Model 4, significantly different from the stability model (Δχ^2^ = 48.518, Δ*df* = 30, *p* = 0.017), fit best with the data.

## Discussion

In the current study, we examined the changes in academic stress and motivation toward PE among Chinese students and the relationship between these variables across time. Most academic stress factors and controlled motivation toward PE decreased significantly while autonomous motivation increased significantly across time. With regards to their relationship, we found that almost all the academic stress factors were positively related to controlled motivation and amotivation, but only pressure from study, workload, and despondency were negatively related to autonomous motivation. We also found a negative prediction of despondency on autonomous motivation and a positive prediction of amotivation on despondency. However, worry about grades was found to be positively related to autonomous motivation and to negatively predict amotivation.

Contrary to the previous findings on long-term change of motivation (Ullrich-French and Cox, 2014), we found an increase in autonomous motivation and decreases in controlled motivation and amotivation within the semester. According to the Self-Determination Theory, autonomous motivation is related to satisfaction of basic psychological needs, such as autonomy and relatedness (Standage et al., 2012; Ryan and Deci, 2017). The increase in self-determined motivation in a short time may be explained by the fact that students get to know their teacher and classmates which improves the fulfillment of the relatedness needed during the semester.

Consistent with previous literature (Xiang et al., 2019), we found that academic stress factors, like pressure from study and workload, declined within the semester. As suggested by Xiang et al. (2019), at the beginning of the semester, students lack time to adapt well from holiday to school lives and academic activities seem difficult to them. Therefore, as time goes on, students tend to experience less academic stress because of knowledge accumulation and good preparation.

A high level of stress, particularly academic stress, has been found to be related to less intention to participate in PA and PE among adolescents (Back, 2015). Similarly, we also found a negative relationship between academic stress and self-determined motivation toward PE. In particular, pressure from study, workload, and despondency was negatively associated with autonomous motivation but positively associated with controlled motivation and amotivation. Nevertheless, worry about grades was positively related to both autonomous motivation and controlled motivation. In previous studies (Sun et al., 2011, 2013), worry about grades, unlike other academic stress factors, was not found to be related to negative consequences, such as low efficacy and poor health condition. Therefore, we suggest that worry about grades may function as one positive source of academic stress.

Both academic stress and motivation toward PE were found to influence each other. A previous study of students from Shanghai also showed that academic burden was perceived as the primary reason for insufficient PA and longer homework time (Zhu et al., 2017). In the current study, we found despondency as a negative predictor of autonomous motivation which suggests that the impact of academic stress on participation in PE may come from the reduced self-determined motivation. However, not all academic stress factors were found to be predictors of reduction of self-determined motivation, especially worry about grades was found to be a negative predictor of amotivation. In a previous study on academic stress and PA, Frömel et al. (2020) also found that girls with academic stress presented low participation in PA during school but high participation in PA after school. Thus, we suggest that some academic stress, such as worry about grades, may not necessarily lead to a negative impact on motivation and/or participation in PE. Concerning the influence of motivation toward PE on academic stress, we found that amotivation positively predicted despondency. Previous studies have shown that participation in PE can be related to positive academic outcomes, such as less academic stress and higher test scores (Sallis et al., 2012; Back, 2015; Shen, 2017). The encountered influence of amotivation on academic stress implies that the impact of PE on academic outcomes may function through the motivation toward PE and academic activities (Torrijos-Niño et al., 2014). However, the influence of autonomous motivation on academic stress was not found significant, which suggests that autonomous motivations may not affect directly academic stress.

### Implications

Since 2002, the Chinese Ministry of Education has conducted PE curriculum reform with a focus on shifting from sports skills to health and fitness development (Jin, 2013). Nevertheless, Chinese adolescents reported increasing physical inactivity and failed to meet the PA guidelines. Prioritized academic achievement over PA in Chinese culture has been one of the barriers to participation in PE (Jones, 1999; Zhu et al., 2017). The influence of academic stress on motivation toward PE found in our study implies that high academic stress levels may reduce participation in PE through decreased self-determined motivation toward PE. On the other side, academic stress has drawn more and more attention from parents, educators, and government, especially in China and other Asian countries (Liu and Lu, 2012). To alleviate stress, PE has been believed to be an effective method (Ki et al., 2019). However, increasing PE sessions does not necessarily guarantee benefits (Resaland et al., 2016). As the traditional PE curriculum in China is designed to promote endurance, strength, flexibility exercises, and circuit training for cardiovascular health, students have little choice in selecting sports during PE courses. The encountered influence of amotivation on academic stress suggests that for students who lack motivation toward PE, being forced to participate in PE may increase rather than decrease academic stress. Taking these together, when designing and administrating interventions to reduce academic stress or to promote PA participation, we recommend taking both principal subjects related to academic factors and PE course into account simultaneously, such as academic burden and quality of PE which have been found to be influential factors of academic stress and PA participation (Zhu et al., 2017; Zhang et al., 2019).

In China, PE is less emphasized at school due to the idea that sports require little intellect and PE is not part of the important entrance examination. A similar view is held by most parents and educators worldwide that the increasing time on PA will be at the expense of decreased time on principal subjects (Wang, 2017). On the contrary, studies have suggested that PE and other PA programs would benefit academic performance (e.g., Sallis et al., 2012; Back, 2015; Shen, 2017). Torrijos-Niño et al. (2014) suggest that the positive impact of PE on the academic achievement of principal subjects may also exert indirectly through motivation. In the current study, academic stress was found to negatively predict motivation toward PE, similar to the negative influence of academic stress on academic motivation found in previous studies (Liu, 2015; Rubach and Bonanati, 2021). Meanwhile, students who lack motivation toward PE also reported despondency in principal subject learning. The negative impact of academic stress and motivation toward PE on each other provides evidence that the impact of motivation-related factors in a certain situation, such as PE, can be generalized to other academic subjects (Ryan and Deci, 2017).

Although various studies have found a negative influence of academic stress on students’ psychological wellbeing and academic performance, academic stress may not always lead to negative consequences. As suggested by some researchers (Sang et al., 2018; You, 2018; Ye et al., 2019), when students view such stress as a challenging opportunity, the academic stress is more likely to result in a positive impact (Sang et al., 2018). Scales et al. (2020) also found that the challenge provided by teachers (high expectations) as an element of developmental relationships had a positive influence on students’ academic motivation and sense of belonging. The negative prediction of worry about grades on amotivation toward PE confirms the positive influence of academic stress. When academic stress comes from high expectations which students may perceive as a challenge rather threat, the positive impact of academic stress can also be generalized to other contexts. Accordingly, providing challenges conforming to students’ abilities may be an effective method to enhance students’ motivation for both principal academic subjects and PE courses.

### Limitations and future research

The most important limitation of this study is that we conducted the study in specific Chinese culture, so we need to be cautious when generalizing and interpreting some of the results to other cultures. Although the idea that the increasing time on PE will lead to decreased time on principal subjects and poor academic performance is held by parents and educators worldwide (Wang, 2017), the over-emphasis on academic performance in Chinese culture may make students more sensitive to academic stress and its influence. We suggest conducting similar research in other cultures to examine whether such a reciprocal relationship between academic stress and motivation toward PE is consistent across different cultures.

Second, we only measured academic stress and motivation toward PE within a semester. Previous studies have shown that both academic stress and motivation vary across semesters and grades in different ways (Ullrich-French and Cox, 2014; Barker et al., 2018). Future research will need to measure both variables within and across semesters to investigate how academic stress and motivation toward PE affect each other in both the short term and long term.

Finally, the encountered positive relationship between worry about grades and autonomous motivation reveals that not all academic stress factors have a negative impact on students’ participation in PE. To examine the possible positive influence of academic stress on students’ wellbeing, it would be useful to include consequent variables, such as intention to participate in PE, PA level during PE, and positive and negative affect, which are highly correlated with academic stress and motivation toward PE (Torrijos-Niño et al., 2014). In addition, as both academic stress and motivation would be influenced by parenting style and teaching styles (e.g., Van Der Kaap-Deeder et al., 2019; Moè et al., 2020), we also recommend including variables such as autonomy support and psychological control to further explore the relationship between academic stress and motivation.

## Conclusion

Similar to previous studies on the relationship between academic stress and academic motivation, we found a reciprocal relationship between academic stress and motivation toward PE. The current study provides evidence to support that the influence of motivation-related factors can be generalized to other contexts, as suggested by Ryan and Deci (2017). Specifically, the influences of despondency and self-determined motivation on each other suggest that academic stress may hinder participation in PE through reduction of self-determined motivation while lack of motivation toward PE may lead to an increase of academic stress through the generalized impact of motivation in PE to other educational situations in the school context. Therefore, whether to relieve students’ academic stress or to promote PA participation, educators and administrators should take academic factors related to principal subjects and PE courses together. Finally, the positive relationship between worry about grades and self-determined motivation toward PE suggests that providing students adequate challenge as a source of academic stress may enhance their self-determined motivation not only in a principal subject-related academic context but also in a PE context.

## Data availability statement

The datasets presented in this study can be found in online repositories. The names of the repository/repositories and accession number(s) can be found below: https://osf.io/dtmnk/?view_only=7b80b0b6fbe349e8aa1f7b0ca149a0fd.

## Ethics statement

The studies involving human participants were reviewed and approved by East China Normal University. Written informed consent to participate in this study was provided by the participants’ legal guardian/next of kin.

## Author contributions

MY contributed to the data collection. MY and CV analyzed the data and approved the final manuscript. All authors conceived the hypothesis of this study and drafted and/or revised the manuscript.
